# Supplementary material for: Changes in HbA1c during the first six years after the diagnosis of Type 2 diabetes mellitus predict long-term microvascular outcomes
Source: PLoS One. 2019 Nov 27;14(11):e0225230. doi: 10.1371/journal.pone.0225230 (PMC6881005; doi:10.1371/journal.pone.0225230)
Supplement: S1 Table — a The Danish National Death Registry and the National Hospital Discharge Registry changed coding from International Classification of Diseases 8 (ICD-8) to ICD-10 on 1 January 1994. The Danish National Death Registry contains only the first 4 characters of the ICD codes, while the National Hospital Discharge Registry contains all 5 characters. b The National Hospital Discharge Registry changed coding of surgical procedures from the 3rd edition of The Danish Classification of Surgical Procedures to the Nordic Classification of Surgical Procedures on 1 January 1996. (DOCX) [file pone.0225230.s001.docx]

**S1 table. Definition of clinical outcomes in the Diabetes Care in General Practice (DCGP) 19-year registry-based monitoring used to classify cause of death or morbidity**.

|  | **Codes to classify cause of death or morbidity** | | **Codes to classify surgical procedures** | |
| --- | --- | --- | --- | --- |
|  | ICD-8 codes (≤1993)^a^ | ICD-10 codes (≥1994) ^a^ | The Danish Classification of Surgical Procedures ((≤1995) ^b^ | The Danish Classification of Surgical Procedures ((≤1996) ^b^ |
| **Any diabetes-related endpoint** |  |  |  |  |
| Sudden death | 795 or 796.2 or 796.3 or 796.9 | R96-R99 |  |  |
| Death from hyperglycaemia |  | E10.0 or E10.1 or E11.0 or E11.1 or E12.0 or E12.1 or E13.0 or E13.1 or E14.0 or E14.1 |  |  |
| Death from hypoglycaemia |  | E16.0 or E16.1 or E16.2 |  |  |
| Fatal myocardial infarction | 10-414 or 427.0 or 427.1 or 427.9 or 428 | I20-I25 or I50 |  |  |
| Nonfatal myocardial infarction | 410 | I21 |  |  |
| Angina/ischaemic heart disease | 411-414 | I20 or I25 |  |  |
| Heart failure | 427.0 or 427.1 or 427.99 or 428 | I50 |  |  |
| Fatal stroke | 430-438 | I60-I69 or G45 |  |  |
| Nonfatal stroke | 430-434 or 436 | I60-I64 |  |  |
| Nonfatal renal failure | 792% | N18-N19% | 94340 or 94300 | TJA 30 |
| Fatal renal disease | 580-593 | N00-N06 or N08 or N10- N13 or N17- N20 or N25- N29 or E10.2 or E11.2 or E12.2 or E13.2 or E14.2 |  |  |
| Amputation |  |  | 8103 or 8104 or 8105 or 81080 or 81081 | NEQ or NFQ or NGQ or NHQ |
| Fatal peripheral vascular disease | 440.2 | I70.2 or E10.5 or E11.5 or E12.5 or E13.5 or E14.5 |  |  |
| Vitreous haemorrhage | 377.00 | H43.1 or H45.0 | 16540 | CKD65 |
| Retinal photocoagulation |  |  | 16070 | CKC10 or CKC15 |
| Blindness | 379 | H54.0 or H54.1 or H54.4 |  |  |
| Cataract excision |  |  | 17000 or 171 or 1720 or 1721 or 1723 or 1726 | CJC or CJD or CJE |
| **All-cause mortality** | All codes | All codes |  |  |
| **Diabetes-related mortality** |  |  |  |  |
| Fatal myocardial infarction | 10-414 or 427.0 or 427.1 or 427.9 or 428 | I20-I25 or I50 |  |  |
| Fatal stroke | 430-438 | I60-I69 or G45 |  |  |
| Renal disease | 580-593 | N00-N06 or N08 or N10- N13 or N17- N20 or N25- N29 or E10.2 or E11.2 or E12.2 or E13.2 or E14.2 |  |  |
| Hyperglycaemia |  | E10.0 or E10.1 or E11.0 or E11.1 or E12.0 or E12.1 or E13.0 or E13.1 or E14.0 or E14.1 |  |  |
| Hypoglycaemia | 962.3 | E16.0 or E16.1 or E16.2 |  |  |
| Sudden death | 795 or 796.2 or 796.3 or 796.9 | R96-R99 |  |  |
| Peripheral vascular disease | 440.2 | I70.2 or E10.5 or E11.5 or E12.5 or E13.5 or E14.5 |  |  |
| **Myocardial infarction** |  |  |  |  |
| Fatal myocardial infarction | 410-414 or 427.0 or 427.1 or 427.9 or 428 | I20-I25 or I50 |  |  |
| Nonfatal myocardial infarction | 410 | I21 |  |  |
| **Stroke** |  |  |  |  |
| Fatal stroke | 430-438 | I60-I69 or G45 |  |  |
| Nonfatal stroke | 430-434 or 436 | I60-I64 |  |  |
| **Peripheral vascular disease** |  |  |  |  |
| Amputation |  |  | 8103 or 8104 or 8105 or 81080 or 81081 | NEQ or NFQ or NGQ or NHQ |
| Death from peripheral vascular disease | 440.2 | I70.2 or E10.5 or E11.5 or E12.5 or E13.5 or E14.5 |  |  |
| **Microvascular disease** |  |  |  |  |
| Nonfatal renal | 792 | N18-N19 | 94340 or 94300 | TJA 30 |
| Fatal renal | 580-593 | N00-N06 or N08 or N10- N13 or N17- N20 or N25- N29 or E10.2 or E11.2 or E12.2 or E13.2 or E14.2 |  |  |
| Vitreous haemorrhage | 377.00 | H43.1 or H45.0 | 16540 | CKD65 |
| Retinal photocoagulation |  |  | 16070 | CKC10 or CKC15 |

^a^ The Danish National Death Registry and the National Hospital Discharge Registry changed coding from International Classification of Diseases 8 (ICD-8) to ICD-10 on 1 January 1994. The Danish National Death Registry contains only the first 4 characters of the ICD codes, while the National Hospital Discharge Registry contains all 5 characters.

^b^ The National Hospital Discharge Registry changed coding of surgical procedures from the 3rd edition of The Danish Classification of Surgical Procedures to the Nordic Classification of Surgical Procedures on 1 January 1996.
